# Imaging of dynamic actin remodeling reveals distinct behaviors of head and trunk mesoderm in gastrulating *Xenopus* *laevis*

**DOI:** 10.17912/micropub.biology.000483

**Published:** 2021-10-14

**Authors:** Valerie Komatsu, Viraj Doddihal, Chenbei Chang

**Affiliations:** 1 University of Southern California, Los Angeles, CA 90089; 2 Stowers Institute for Medical Research, Kansas City, MO 64110; 3 University of Alabama at Birmingham, Birmingham, AL 35294; 4 Embryology: Concepts & Techniques in Modern Developmental Biology, Marine Biological Laboratory, Woods Hole, MA, 02543

## Abstract

Gastrulation involves coordinated movements of cells, facilitating mesoderm and endoderm internalization and proper patterning of tissues across the germ layers. In *Xenopus laevis*, head mesoderm migrates collectively along the blastocoel roof fibronectin network towards the animal pole. Meanwhile, the trunk mesodermal cells migrate over each other in convergent thickening and convergent extension movements elongating the body axis. The behaviors of cells in these regions are investigated mainly in tissue explants taken from the respective head or trunk mesodermal regions. How cells behave at the transitional zone between these territories is not described in detail. To learn about cell behaviors around this junction, we imaged cell movements in an explant that encompassed the head and trunk mesoderm. We observed that head mesoderm migration on fibronectin employed lamellipodial protrusions at the leading edge and dynamic actin remodeling in the trailing cells. Trunk mesodermal cells underwent mediolateral cell elongation and intercalation to form the notochord. Lateral edges of the notochord were defined before the anterior edge. Our movie reveals distinct mesodermal cell behaviors occurring simultaneously in different regions of gastrulating embryos. This study highlights the power of applying modern microscopy tools to revisit classical experiments, permitting a greater understanding of the cellular dynamics that shape the embryo.

**Figure 1. Time-lapse microscopy of head mesoderm migration and notochord segregation and intercalation in Xenopus explant:**
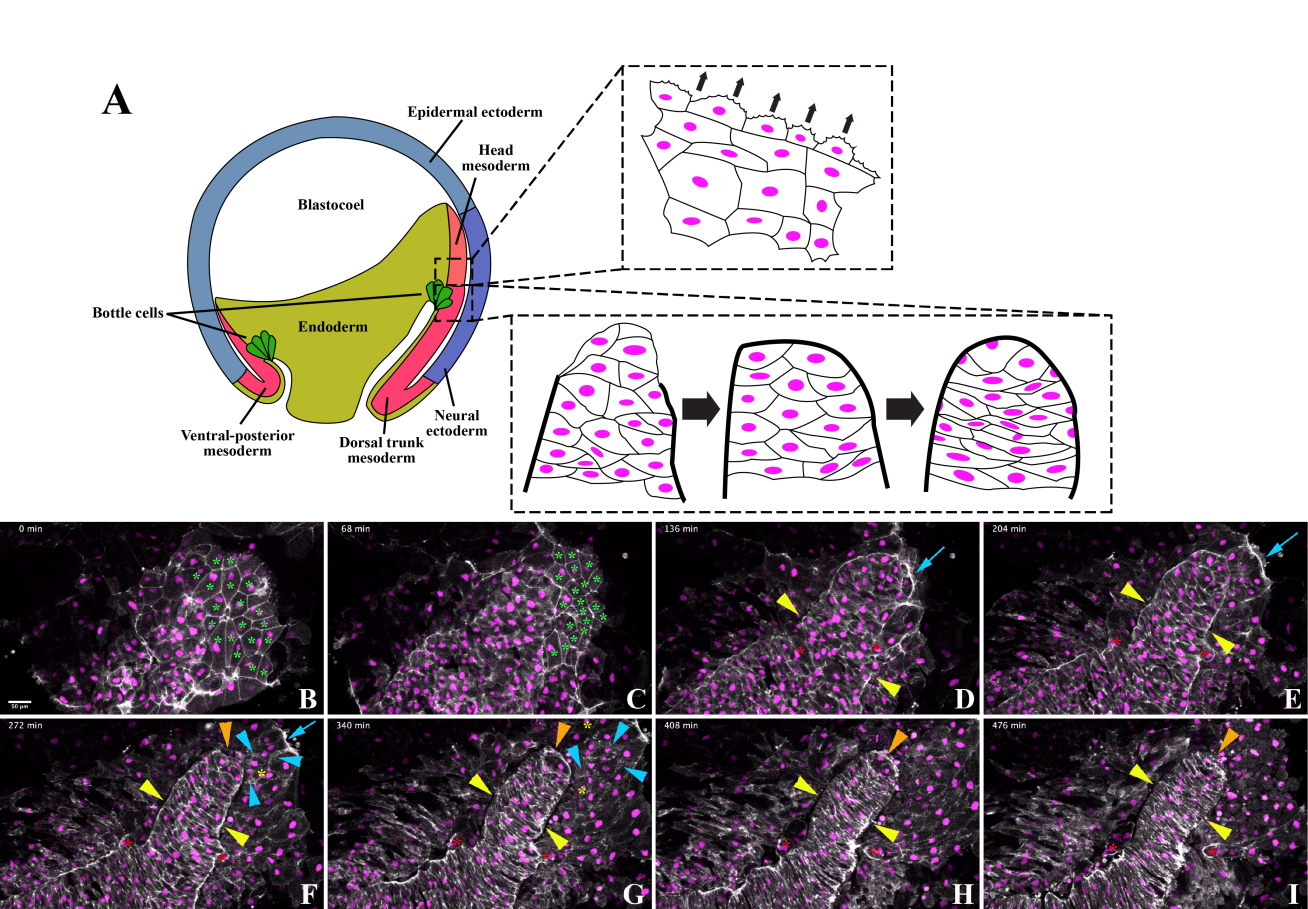
A) Diagram of the embryonic region used in this experiment and a summary of observed cell behaviors. Early *Xenopus laevis* embryos were injected with mRNAs encoding LifeAct-GFP and histone H2B-RFP. Dorsal tissues in the anterior region of the post-involuted mesoderm from mid-gastrula embryos (dotted box) were taken for time-lapse microscopy for a total of 8 hours with frame intervals of 4 minutes. Head mesoderm cells exhibited F-actin polarization at the leading edge of the migration front, forming distinctive lamellipodia (top panel). The notochord formed from the trunk mesoderm, first forming lateral borders defined by high intensity F-actin signals, while the anterior border formed later (bottom panel). Cells within the notochord elongated and underwent convergent extension to straighten and extend the notochord. B-I) Selected maximum intensity projections from the movie are shown. The anterior-posterior axis is diagonal from the upper right to the lower left. The starting frame at 0 minutes (min) is shown in panel B, and subsequently every 17th frame is shown in panels C to I. The grey and magenta pseudocolor are the F-actin (LifeAct-GFP) and cell nuclei (H2B-RFP) signals, respectively. The green asterisks in panels B and C indicate the endodermal cells that are pushed out of the focal plane by the advancing mesodermal cells. The yellow and the orange arrowheads point to the lateral (panels D to I) and the anterior (panels F to I) notochordal boundaries, respectively. The blue arrows in panels D to F point to the lamellipodia in the leading-edge migrating head mesodermal cells. The blue arrowheads in panels F and G point to some examples of dynamic F-actin in protrusions in the trailing head mesodermal cells. The red asterisks in panels D to I highlight two cells that moved from their initial positions within the notochordal boundaries into the adjacent somatic region. The yellow asterisks in panels F and G indicate examples of new nuclei that appeared between migrating head mesodermal cells.

## Description

To visualize actin dynamics, cell shape changes, and cell movements during *Xenopus laevis* gastrulation, we injected mRNAs encoding LifeAct-GFP (Shindo *et al.*, 2019) and histone H2B-RFP (Butler and Wallingford, 2018) into early *Xenopus* embryos. At the mid-gastrula stages, we dissected the dorsal tissues around the anterior region of the post-involuted bottle cells (Fig. 1A). We then placed the explants with the endodermal side down on fibronectin-coated glass-bottom dishes. Time-lapse microscopy was performed on an inverted confocal microscope. We captured the movements of the head and trunk mesodermal cells, visualizing distinct behaviors in the two regions (Fig. 1A). Below, we describe in more detail a movie that documented head mesoderm migration and notochord segregation in these regions. Cell movements were recorded in an explant for 8 hours with a capture interval of 4 minutes (Extended Data Movie 1).

We observed prominent migration and dynamic actin reorganization of the head mesodermal cells. The polygonal endodermal cells, defined by stable F-actin signal at cell-cell junctions (Fig. 1B and C, green asterisks), were pushed out of the focal plane by a cohesive group of cells exhibiting less-defined junctional F-actin but more active protrusions (t = 0-140 min in Extended Data Movie 1 and Fig. 1B and C). The cells in the anterior region actively spread outwards, displaying a concentrated F-actin signal at the leading edge where we observed dynamic lamellipodia (t= 148-232 min in Extended Data Movie 1; blue arrows in Fig. 1D to F). We also detected F-actin clusters and protrusions in the trailing migratory cells, demonstrating that these cells undergo active actin remodeling, potentially assisting with migration (Fig. 1F and G, blue arrowheads). However, we did not identify significant polarity of F-actin in the trailing cells.

The F-actin behaviors we captured reveal novel insights into head mesoderm migration. Previous work in fixed samples and whole tissue explants have implicated both leading and trailing cells in assisting with collective cell migration (Damm and Winklbauer, 2011; Nagel *et al.*, 2021; Winklbauer and Nagel, 1991). While our movie demonstrates that leading cells extend protrusions that may drive collective cell migration, we did not observe F-actin polarization in trailing cells. A lack of F-actin polarization in trailing cells suggests that head mesoderm migration does not require directional cues secreted from the blastocoel roof, which is absent in our explant. Blastocoel roof-secreted directional cues were required however for the persistent spreading of mesodermal cells, as the cells seemed to retract by the end of the 8-hour time-lapse movie. Mesodermal cell retraction suggests that endogenous signals, such as those from the chemoattractants PDGF (platelet-derived growth factor) and SDF-1 (stromal cell-derived factor 1), may be required to maintain persistent migration of the head mesoderm (Ataliotis *et al.*, 1995; Damm and Winklbauer, 2011; Fukui *et al.*, 2007; Nagel *et al.*, 2004; Symes and Mercola, 1996). These are hypotheses that can be explored in future experiments.

We observed migration events that may elucidate the mechanism of head mesoderm thinning. The head mesoderm rapidly flattens through PDGF-dependent radial intercalation (Damm and Winklbauer, 2011). Previous data have shown that mesodermal cells deep within the embryo radially intercalate with more superficial head mesodermal cells, potentially driving thinning (Winklbauer and Nagel, 1991). However, F-actin dynamics of these cells have not been reported. We observed new cell nuclei between head mesoderm migratory cells, capturing their F-actin dynamics (Fig. 1F and G, yellow asterisks). These cells may correspond to the aforementioned deep mesodermal cells that drive head mesoderm thinning. Our dataset, in combination with future experiments in similar explants, could assess the role of F-actin polarization in head mesoderm thinning.

In contrast to the head mesoderm, we observed notochord morphogenesis through border formation and cell intercalation in the trunk region. We identified the notochordal boundary through a concentrated F-actin signal that segregated the notochord from surrounding cells. Enhanced F-actin intensity delineating tissue borders became discernable at 80 minutes and apparent at 160 minutes into imaging (Extended Data Movie 1 and Fig. 1B to H, yellow arrowheads). While the lateral notochord boundary formed first, cells continued to be added into the anterior notochord until about 280 minutes into imaging (Extended Data Movie 1). The notochord formed a distinctive anterior boundary 288 minutes into imaging, identifiable by continuous, high-intensity F-actin signal (Extended Data Movie 1 and Fig. 1F to H, orange arrowheads). These observations are consistent with previous work demonstrating that the lateral notochord boundary is defined prior to the anterior border (Youn *et al.*, 1980). Some cells within the nascent notochord were expelled despite these apparent boundaries, relocating to paraxial positions adjacent to the definitive notochord (red asterisks for two such cells in Fig. 1D to 1I). Expulsion of notochord mesodermal cells has been described previously (Reintsch *et al.*, 2005; Shih and Keller, 1992). Initially, the cells that remained within the lateral boundaries of the notochord formed a column 3 to 5 cells wide. These cells underwent mediolateral elongation, during which the anterior (A) and posterior (P) cell contacts expanded while mediolateral cell contacts were reduced. Mediolateral elongation resulted in the notochord cells intercalating among themselves, extending the presumptive notochord without adding additional mesodermal cells (Extended Data Movie 1 and Fig. 1). Notochord cell intercalation and elongation commenced before the appearance of the high intensity F-actin signal that delineates the anterior notochordal border (Fig. 1F to I, orange arrowheads). The position of cell nuclei did not alter drastically during notochord mediolateral elongation, suggesting that intercalation was mediated mainly by cell shape changes rather than cell crawling and translocation (Extended Data Movie 1). The notochord narrowed and straightened as intercalation proceeded, especially in the posterior region (Extended Data Movie 1 and Fig. 1F to I). As the cells elongated and intercalated, the F-actin intensity at the notochordal boundary was reduced, but the F-actin signal at the A-P cell contacts remained strong (Fig. 1I). Eventually, a stack of elongated cells was seen spanning the notochord (Fig. 1I). The trunk mesodermal cell behaviors observed agree with those described previously (Keller *et al.*, 1989; Wilson and Keller, 1991; Wilson *et al.*, 1989) and are regulated by Wnt/planar cell polarity signaling (Moon *et al.*, 1993; Wallingford *et al.*, 2000).

Our movie reveals distinct behaviors in migrating head and intercalating trunk mesodermal cells in the same explant. For both regions, we detected very few cell divisions during morphogenesis but observed distinctive F-actin dynamics (Extended Data Movie 1). While some cell and cytoskeleton behaviors have been reported previously using different types of explants or fixed and sectioned embryos (Fagotto *et al.*, 2013; Keller *et al.*, 1989; Shih and Keller, 1992; Winklbauer and Selchow, 1992), this is the first study to our knowledge that examines F-actin dynamics at the head-trunk mesodermal interface in live explants. Our live imaging contributes new insights about F-actin behaviors in migrating head mesoderm that complements previous studies on membrane protrusive activities in these cells (Nagel *et al.*, 2021; Winklbauer and Nagel, 1991). Our study also reveals temporal dynamics of notochordal boundary formation that was implicated by scanning electron microscopy studies, but never before shown in live explants (Youn *et al.*, 1980). Explants that include both the head and trunk mesoderm can be further used in future studies to explore how cells coordinate their behaviors during gastrulation (Hara *et al.*, 2013). Our study demonstrates that recapitulating classical experiments using modern techniques can not only confirm previous findings but reveal unique perspectives into the fundamental processes that govern early embryonic development.

## Methods

The *Xenopus laevis* frogs were used in accordance with the animal usage protocol 21-06A approved by the IACUC committee at the Marine Biological Laboratory (MBL) for the Embryology course. The embryos were obtained by in vitro fertilization. A mixture of RNAs encoding LifeAct-GFP (Shindo *et al.*, 2019) and H2B-RFP (Butler and Wallingford, 2018) was injected at the 100pg dose each into early embryos. The explants were dissected at mid-gastrula stage 11.5 in the DFA solution by taking the dorsal tissues surrounding the post-involuted bottle cells marked by the distinct black line in the anterior region of the archenteron. The explants were mounted in the DFA solution with the endodermal side facing down on a glass-bottom dish pre-coated with 100 μg/ml fibronectin at room temperature for 30 minutes. Time-lapse microscopy was performed on Zeiss LSM 780 laser scanning microscope using a 20x objective at 0.6x zoom with the following setup: image resolution of 512 x 512 pixels, z-stack with 0.871 μm step size for a total of 54 z-axial steps, and a time lapse with an interval of 4 minutes for a duration of 8 hours. A selected set of the still images of maximum intensity projections are shown in Figure 1. The entire movie is included in the appended material.

## Reagents

DFA (Danilchik for Amy) solution:

49.5mM NaCl

36.5 mM gluconic acid, sodium salt

5 mM Na2CO3

4.5 mM KCl

1 mM CaCl2

1 mM MgSO4

1 x antibiotic

0.1% BSA

Adjust to pH 8.1 with HEPES

Filter sterilize.
